# The care manager meeting the patients’ unique needs using the care manager model—A qualitative study of experienced care managers

**DOI:** 10.1186/s12875-021-01523-8

**Published:** 2021-09-03

**Authors:** Irene Svenningsson, Dominique Hange, Camilla Udo, Karin Törnbom, Cecilia Björkelund, Eva-Lisa Petersson

**Affiliations:** 1 Primary Health Care/Department of Public Health and Community Medicine, Institute of Medicine, Sahlgrenska Academy, University of Gothenburg, Gothenburg, Sweden; 2 Research, Education, Development & Innovation, Primary Health Care, Region Västra Götaland, Sweden; 3 School of Education, Health and Social Studies, Dalarna University, Falun, Sweden; 4 Center for Clinical Research, Dalarna, Sweden; 5 Department of Social Work, University of Gothenburg, Gothenburg, Sweden

**Keywords:** Care manager, Collaborative care, Common mental disorder, Nurses, Primary health care, Qualitative study

## Abstract

**Background:**

Implementation of a care manager in a collaborative care team in Swedish primary care via a randomized controlled trial showed successful outcome. As four years have elapsed since the implementation of care managers, it is important to gain knowledge about the care managers’ long-term skills and experiences. The purpose was to examine how long-term experienced care managers perceived and experienced their role and how they related to and applied the care manager model.

**Method:**

Qualitative study with a focus group and interviews with nine nurses who had worked for more than two years as care managers for common mental disorders. The analysis used Systematic Text Condensation.

**Results:**

Four codes arose from the analysis: Person-centred; Acting outside the comfort zone; Successful, albeit some difficulties; Pride and satisfaction. The care manager model served as a handrail for the care manager, providing a trustful and safe environment. Difficulties sometimes arose in the collaboration with other professionals.

**Conclusion:**

This study shows that long-term experience of working as a care manager contributed to an in-depth insight and understanding of the care manager model and enabled care managers to be flexible and act outside the comfort zone when providing care and support to the patient. A new concept emerged during the analytical process, i.e. the *Anchored Care Manager*, which described the special competencies gained through experience.

**Trial registration:**

NCT02378272 Care Manager—Coordinating Care for Person Centered Management of Depression in Primary Care (PRIM—CARE).

## Background

The majority of patients with common mental disorders (CMD), including anxiety syndrome, depression and stress-related mental disorder, are treated within primary health care (PHC) [1]. To meet this group of patients, new ways of team-based working have been developed. The Collaborative Care Model (CCM) is one such approach that has been established [2]. CCM is based on Wagner’s Chronic Care Model [3], which includes four key criteria: team-based approach to patients, structured care plan, scheduled patient follow-ups, and enhanced inter-professional communication [4, 5].

The CCM is found to be cost effective for physical and mental health [6]. Compared to usual care, CCM has been shown to be effective for anxiety and depression [4]. In a randomized controlled trial, Gilbody et al. compared collaborative care with usual care of elderly people and found decreased depressive symptoms in the short term [7].

Patient care can be improved by a care manager model within a CCM intervention [8].

The care manager puts the collaborative care model into practice by combining the responsibility for providing support, accessibility and continuity to the individual patient. This is a complex intervention that requires broad knowledge about individuals with CMD, as well as communication and organisational skills. This complex intervention also includes the physician and other professionals in the care team [8, 9].

The cornerstone of care is the initial person-centred meeting incorporating the patient’s concerns and expectations. On the basis of this discussion, the patient’s needs are combined with the best available resources [9].

The care manager is usually a specially educated nurse who supports the CMD patient in self-care during a period of three months [10]. The care manager meets the patient at the PHC in a face-to-face consultation at first, with follow-ups by telephone [8]. Telephone follow-ups have been shown to be a good tool for monitoring patients with CMD [11] and have been found to decrease the depression symptoms [12]. The care manager follows the patient’s symptoms and provides information and support regarding medical and psychological treatment options. The patient and the care manager agree in advance to a care plan based on a person-centred approach [8]. Involving the patient as an equal partner and an expert improves health outcomes and increases patient satisfaction [13]. The care manager role has been described in detail in an earlier study [8].

The change to the CCM organisation with a care manager in primary health care is a complex intervention. The implementation requires modified educational programmes for care management, good systems for communication and monitoring, reimbursement schemes, interaction between care manager, general practitioners and other professionals and co-location, preferably at the PHC [14, 15]. Implementation of a care manager in a collaborative care team in Swedish primary care via a randomized controlled trial showed successful outcomes in terms of decreased depression symptoms, improved quality of life and increased return to work in a three months perspective [8]. The care manager model has now been implemented in most PHCs in one region of Sweden and has been expanded to include patients with CMD diagnoses such as anxiety and stress-related disorders. According to results from a focus group study with newly educated care managers, care managers perceived improved continuity, accessibility and a meaningful and empowering way of working [16]. As four years have elapsed since the implementation of care managers in primary health care, it is important to gain knowledge about the care managers’ long-term skills and experiences and how care managers relate to the care manager model in daily practice.

## Methods

### Aim

The purpose was to examine how long-term experienced care managers perceived and experienced their role and how they related to and applied the care manager model.

### Design

This is a qualitative study using data from both focus group discussion [17, 18] and individual interviews [19]. We used Malterud’s [20] Systematic Text Condensation (STC) for the analysis.

### Setting and participants

From the 100 PHCs operated by private and public actors in urban and rural areas in one region of Sweden, one care manager at each PHC was potentially eligible for participation. Among these, invitations to participate were extended to care managers who had worked for more than two years as a care manager. Thus, all 22 RNs working as care managers for more than two years were invited to participate in the study. The invitation was orally forwarded to all care managers at a yearly information meeting, after which 12 persons announced an interest in participation. Of these 12 persons, three could not participate due to lack of time, leaving nine care managers who were included. The participants, eight women and one man, had worked as nurses for between seven and 35 years.

We conducted one focus group (*n* = 5) and four individual interviews. All participants provided written consent indicating that they were informed about the purpose of the study and that participation was voluntary. The focus group discussion and the interviews were audio-recorded, transcribed verbatim, and kept confidential. The duration of the focus group was 60 min and the duration of the interviews was 30–60 min. Both procedures took place at a conference centre.

### Data collection

The focus group discussion was moderated by ELP with IS as assessor. The focus group guide was based on the following questions: What is it like to work as a care manager? How do you experience your role as a care manager at the PHC? What role does the care manager have at the PHC? The individual interview guide was based on the following questions: How do you work as a care manager? In what way do you provide support to patients? How do you follow up the patients? Are there difficulties in the working model? What is included in the role of the care manager at the PHC? With whom do you collaborate at the PHC? Are you part of a team at the PHC as a care manager? In your role as care manager, do you have any influence on the organisation regarding patients with poor mental health at the PHC?

The participants discussed their experiences of working as care managers. We had originally planned to do two focus groups, but due to the participants’ personal commitments, one focus group was cancelled. Instead, we conducted individual interviews. The individual interviews were conducted by ELP and IS. ELP is an occupational therapist and IS is a district nurse; both are associate professors and have long experience from primary health care. The focus group and the interviews took place in spring and autumn 2019.

### Analysis

The analysis was conducted according to STC by Malterud [20]. The data from the focus group and the individual interviews was merged into one data set in the analysis, and no differentiation was made between data obtained from the focus group and the individual interviews.

The analysis was data-driven, with no theoretical framework as template. The following four steps guided the analysis: 1) To obtain an overall impression, the data set was read several times to get a general sense of the whole statement. 2) Meaning units were then identified with focus on care managers’ experiences and coded. 3) The content of the subgroups from each code were sorted and reduced into a condensate—an artificial quotation maintaining the original terminology applied by the participants. 4) The content of each code group was summarised into a general description. See the analytical process (Table 1).

**Table 1:** Description of the analytical process

Meaning Units	Code group	Subcode
These feelings of trust and safety are achieved after having met the patient for a while. Because they have difficult issues to share and to speak about	Person-centred	Trust
The most important value for the patients, as I see it, is that; “I am not alone in this”. I say this over the phone; “we will do this together”. You shouldn’t sit at home, wondering about this		We will do this together
I thought it was really stressful to document everything in an hour. So, I’ve changed that. Another thing is that some patients don’t want to show up in person for the last visit. If they want to take it over the phone instead, I try to make that happen	Acting outside the comfort zone	Avoided a stressful situation
When you can stay with the patient for longer, this will entail something. And it will challenge your own role as well; “what can I fill this extra time with when I’m allowed to stay for a little longer?”		Challenge the care manager role
So, we allocate the patients based on the guidelines, and as registered professionals we have the right to see patients based on what they seek care for. If the patient presents with anxiety, it’s not obvious that they should visit the GP	Successful, albeit some difficulties	Specified care
These patients with complex issues and a lot of various problems, they need a lot of help. It’s really good when you can sit down together and discuss these cases, and when everyone helps out		Complex issues

The analysis was a collaborative effort involving all co-authors (IS, ELP, KT, CU, CB and DH). CU and KT are social workers with doctoral degrees. CU is an experienced medical social worker meeting patients and families with mental health issues and in crisis. KT has experience from psychotherapy in outpatient care. CB is a general practitioner and professor, and DH is a general practitioner and associate professor. To ensure that the authors’’ preconceptions would not affect the analytical process, reflexivity was used continuously throughout the process [20].

## Results

Four codes emerged from the analysis: Person-centred; Acting outside the comfort zone; Successful, albeit some difficulties; Pride and satisfaction.

### Person-centred

The care manager role was mainly understood as supportive and caring, although limited to a certain time period. Participants wanted to make sure that their patients were personally cared for and wanted to prevent them from feeling abandoned or left alone with unanswered questions.

*The most important value for the patients, as I see it, is that; “I am not alone in this”. I say this over the phone; “we will do this together”. You shouldn’t sit at home, wondering about this* (Interview (I)1)*.*

The direct support that was given mostly concerned advice on how to maintain healthy sleeping, a healthy diet, physical activities, relaxation techniques and continuity in medication. Patients were also encouraged to engage in activities of their own liking and to find a balance in their lives. Follow-ups mainly dealt with how the patient thought about the above-mentioned areas or other personal issues that they considered important for their recovery.

The care managers described how they tried to figure out each patient’s needs, in order to provide valuable advice for how patients could enhance their self-care. Using their experience and knowledge in this consulting way was said to be appreciated by the patients.

To work as a care manager required being updated on the patient’s “personal story”, both in order to form a plan on how to proceed, but also to build a trusting relationship. Creating continuous and trusting relationships was seen as an essential condition for making the patients feel safe enough to share difficult issues about their lives.

*These feelings of trust and safety are achieved after having met the patient for a while. Because they have difficult issues to share and to speak about* (Focus group (FG)).

The care manager provided patients with a more accessible care, and this together with the trust and continuity that the role created was described as major advantages with the care manager function. Participants stressed that new patients could receive more rapid assistance through care managing, and that patients could keep in touch with the same care manager in case they felt worse after their eligible three months.

Participants said that on the one hand, they wanted to be emotionally engaged in their patient’s stories, but on the other hand this was considered both demanding and tiring, especially when compared to their ordinary nursing role which they felt more content with.

*I think it’s a different kind of talk. It’s not a health-talk that you have with some patients, nor is it like continuously dressing a wound… I think the care-manager- talks are heavier* (I 2).

All care managers stressed that the most difficult examples of this were associated with having to care for patients with too severe illnesses, whom they thought belonged to psychiatry.

### Acting outside the comfort zone

Working as a care manager required the ability to understand each patient’s various needs. Participants said that the boundaries for their assignments were flexible enough, allowing them to see the broader picture, to think outside the comfort zone and to work according to their own priorities, the anchored care manager. It was clear that some patients wanted to visit the care manager more frequently or needed a great deal of emotional support, whereas others felt that a phone call every second week was enough.

In addition, participants were more comfortable with the care manager role after having adapted it in ways that better suited themselves and their patients.

*I thought it was really stressful to document everything in an hour. So, I’ve changed that. Another thing is that some patients don´t want to show up in person for the last visit. If they want to take it over the phone instead, I try to make that happen* (I 2)*.*

Participants stressed that the care manager role came with additional patient-time, which gave them opportunities to develop their skills and to do more for the patients.

*When you can stay with the patient for longer, this will entail something. And it will challenge your own role as well; “what can I fill this extra time with when I’m allowed to stay for a little longer?”* (I 1).

An extended room for dialogue with patients was considered highly important in times when patient-time was not considered to be a priority at the primary care centres.

Although the care managers wanted to feel free when planning and executing their work, they praised the clear structure that was achieved when the model was followed. Working according to a clear model made them surer of having provided a safe and professional health care that was equal for all patients.

Participants emphasised that the self-assessment instruments MADRS-S and GAD-7 were valuable in monitoring the patients’ current situation or progress and used them as basis for discussions about what efforts to pursue next. Some participants used a template for documentation that had been provided for the care managing assignments, to get an even more structured documentation. Some participants also used the template, during the patient meeting, to clarify the patient's situation. The care managers used the templates in different ways.

*The template works well to create a structure, making sure that you cover everything. Some (patients) just keep talking so that you lose track, and the talk ends up being off topic, leaving you without the right information. Then you have the template to check that you cover what you need* (I 4).

Participants appreciated the regular meetings they had with other care managers. As they sometimes felt alone with difficult patient cases, it was a relief to confirm their ideas and thinking with others and to ask colleagues for advice. Additionally, sustaining the work through contact with other care managers increased their motivation and helped them keep focusing on patients with CMD on an everyday basis.

### Successful, albeit some difficulties

Working in a psychosocial team was said to have several advantages. Participants believed that it made all professions more aware of patients with CMD, and through the team patients could be given the appropriate type of help at an early stage. The care manager’s work was also said to be easier to accomplish when patients could be discussed with team members.

*So, we allocate the patients based on the guidelines, and as registered professionals we have the right to see patients based on what they seek care for. If the patient presents with anxiety, it’s not obvious that they should visit the GP* (I 1).

An important function for the care managers in the team was to remind colleagues about how to work with patients with CMD, making sure that everyone followed the same modus operandi. Participants also tried to increase the overall knowledge about patients with CMD at the primary care centre, and they saw it as their responsibility to stand up for this patient group.

Close collaboration with other professions was said to create a more secure health care for the patients. Care managers especially wanted a successful collaboration when dealing with patients with complex needs, and several worked closely with the occupational therapists, physiotherapists, psychologists and the GP.

*These patients with complex issues and a lot of various problems, they need a lot of help. It’s really good when you can sit down together and discuss these cases, and when everyone helps out* (FG).

All participants felt that at times they had received patients with a mental illness that they considered too difficult for them to handle. In such cases, they kept contact with the patient until a psychologist or psychotherapist could take over.

Some care managers stressed that there had been some difficulties in convincing other professions of the value and meaning of their work, especially when the care manager function was new. A few still experienced difficulties in collaboration. As an example, one care manager was excluded from a meeting that was about planning new activities for patients with CMD. The care managers did not know how to deal with such situations, but they guessed that some colleagues did not believe in their competence or working methods.

### Pride and satisfaction

Participants mostly enjoyed working as care managers and considered their job as highly meaningful. They experienced feelings of pride, happiness and relief when they saw patients’ improvements. The overall impression was that a vast majority of patients felt better after a while, both according to the patients’ spontaneous feedback and the self-assessments.

*I very much enjoy working as a care manager. I believe that it’s important work, and I’ve received feedback from the patients that it plays an important role for them too* (I 3).

Care managers stressed that another function they had was to support and unburden other professions, especially the GPs. Due to the many benefits that their work contributed with, participants wanted the care manager function to be strengthened and the concept to be deepened.

Care managers felt that it had been especially rewarding to receive positive feedback or gratefulness when patients explained how much their relationship had meant to them. Some participants were happily surprised that their individual conversations or phone calls seemed to have meant so much for the patient to recover.

## Discussion

The purpose was to examine how long-term experienced care managers perceived and experienced their role and how they related to and applied the care manager model.

The main findings showed that working with the structured care manager model provided a trustful and safe environment for the professionals. The participants mainly used the care manager model in their work, but there were individual differences, illustrated in the result section.

The model also provided a clear structure that could be used as a kind of a handrail by the care manager and contributed to increased job satisfaction and pride in their work. However, convincing colleagues within other professions of the value and meaning of using a care manager was described as sometimes difficult.

During the analytical process, a new concept emerged, i.e. the *Anchored Care Manager*, which described the special competencies gained through experience.

*The anchored care manager cared for the CMD patient using a person-centred approach to the care manager model and when needed, acted outside the role of the care manager. This was accomplished by having the courage to act outside the comfort zone, by using the long-term experience gained from meeting many patients with different needs and by securing mutual collaboration with the psychosocial team where the team's various competencies had been utilised. In turn, this generated added value for the patient, leading to a sustainable care manager organisation.*

The nurses perceived their role as care managers as important, since it added in-depth insight and understanding of the complex needs of patients with CMD. According to our findings, by being able to lean on the structure of the care manager model, the care manager experienced courage and self-confidence, both within the professional team and in the contact with the patient, irrespective of the situation. In primary care, it has been suggested that a holistic perspective may provide more meaning for care providers. Poitras et al. highlights the importance of context-specific factors influencing the activities of nurses in primary care settings such as the drive to help the patient, leadership, and clinical experience [21].

The care manager believed that it was necessary to be the patient’s advocate, to give the patient a voice. In this regard, the care manager’s long-term experience and cumulative knowledge was useful. However, the conversation with some patients was perceived as heavy, which could at times have a negative impact on their feelings at work. We believe that this mainly was related to patients with serious illness who are usually treated in psychiatric services and therefore should not be treated in PHCs. To create a safer care for the patient, close collaboration with other professionals was essential. To be a part of the team was an advantage in the care manager’s work. However, difficulties in the collaboration with other professionals did occur and could have a negative effect on the care manager role. Nevertheless, the overall impression was a feeling of pride and satisfaction with the work as care manager when seeing what impact, it had on the patient. It seems as if the nurses adopted the care manager role and integrated it with their professional/personal competences and that they truly became the care manager. All in all, the nurses had a safe and confident way of working as care manager. Our study showed that the model became a handrail to hold onto, giving them the confidence and freedom to become more flexible and act outside the comfort zone when necessary, based on each patient’s unique needs.

The care of the patients was described as being of great importance, and the participants felt confident based on their experience and knowledge [22]. Care managers with long experience seemed to use their dialogue with the patients for gaining the information that was necessary for accomplishing the care managing task. Finding out the patient’s needs is a pre-requisite for being able to provide advice regarding self-care and for developing a person-centred care plan [23]. At the same time, care managers gained experience through their meetings with patients and their collaboration with the team. The discussions with other care managers in regularly held meetings also helped to integrate their experiences into the role [24]. As found previously, such meetings may also counteract emotional stress [25]. The implications of these findings are that team support and professional meetings are essential for the care manager role to be successful. The participants’ long experience as care managers enabled them to see the broader picture and to act outside the comfort zone. The participants felt comfortable to adapt the care manager role to better suit the situation and their patients.

The motivation among the care managers increased when meeting with other care managers. This is in line with Girard et al. [26], who found that the competence of the care manager was important but that also the care managers’ activities and implementing strategies, as well as context specific factors, were ingredients that facilitated the adoption of the role. The structured model was praised, as it made the participants feel more secure about being able to provide equal health care.

Collaboration with other professionals was sometimes perceived as difficult, and the participants felt alone in the management of difficult patient cases. Support from the PHC’s manager and the team is important [15]. When implementing CCM with a care manager, it is important to take into account implementation strategies and context factors [15, 26]. For example, the physical location of the care manager is essential when working in the team [27].

This study shows that collaboration in a team seems valuable for the care manager in work with patients with CMD. Other studies found that barriers affecting collaboration in PHC were the lack of clarity regarding the different roles in the team and general practitioners’ attitudes towards care managers [27, 28]. Regardless of different barriers, the care managers seem to have found their role and enjoy working with this group of patients. Organisational-related aspects also impact the role of the care manager in PHC, such as the workload and the psychosocial complexity of the patient [29]. The composition of the psychosocial team differed among the PHC, which in turn probably also influenced the care manager role. By providing support and shared responsibility, the care manager unburdens the GPs [30].

The care manager felt satisfaction when receiving positive feedback from the patients about the supporting relationships. According to Webster et al. [31], in order to build an efficient collaboration, the most important member in the team is the patient. Care managers who are well acquainted with the patient have an advantage when it comes to building relationships [31], which was also shown in our study.

The participants were surprised that the individual conversation meant so much for patient recovery, which was also seen in a study by Freilich et al. [32]. The caring and engaged care manager seems to be of great importance for the patient with recurrent depression [33]. To strengthen and deepen the role, it is important to give the experienced care manager the opportunity to act as the hub for the new patient groups such as younger individuals and elderly with poor mental health. The role should also include work with prevention at an early stage.

### Strength and limitations

In this study we follow up nurses’ experiences and perceptions of working as care managers, after they had been working for at least two years. The use of both a focus group and individual interviews was not planned but became a strength. The focus group allowed the participants to discuss their experiences and perceptions of working as care managers in further terms while the individual interviews gave the participant the possibility to express sensitive experiences.

One weakness was that ELP and IS both collected the data and took part in the analysis. This potential bias was compensated for when all authors took part in the analysis. Although the number of participants was few, the data provided coherent stories containing abundant and diverse accounts of what we intended to explore. The data was rich, as the participants shared their experiences and perceptions voluntarily and in an eloquent manner.

## Conclusion

Long-term experience of working as a care manager contributed to an in-depth insight and understanding of the care manager model and enabled care managers to be flexible when providing care and support to the patient. When difficulties arose in the collaboration, that might be due to lack of knowledge transfer between care managers and other professionals. The structured model contributed to a trusting and safe environment. During the analytical process, a new concept emerged, i.e. the *Anchored Care Manager*, which described the special competencies gained through experience.

## Data Availability

To protect the participants confidentiality, the data sets generated and analysed during the current study are not publicly available due to Swedish law, but are available from the corresponding author upon reasonable request.

## Abbreviations

CCM: Collaborative care model
CMD: Common mental disorders
PHC: Primary health care
STC: Systematic Text Condensation
